# Vitamin K Status in Adherent and Non-Adherent Patients with Phenylketonuria: A Cross-Sectional Study

**DOI:** 10.3390/nu12061772

**Published:** 2020-06-14

**Authors:** Renata Mozrzymas, Dariusz Walkowiak, Sławomira Drzymała-Czyż, Patrycja Krzyżanowska-Jankowska, Monika Duś-Żuchowska, Łukasz Kałużny, Jarosław Walkowiak

**Affiliations:** 1 Research and Development Center, Regional Specialist Hospital in Wrocław, H. M. Kamieńskiego Street 73a, 51-124 Wrocław, Poland; mozrzymas@wssk.wroc.pl; 2 Department of Organization and Management in Health Care, Poznan University of Medical Sciences, Przybyszewskiego Street 39, 60-356 Poznan, Poland; dariuszwalkowiak@ump.edu.pl; 3 Department of Bromatology, Poznan University of Medical Sciences, Marcelinska Street 42, 60-354 Poznan, Poland; drzymala@ump.edu.pl; 4 Department of Pediatric Gastroenterology and Metabolic Diseases, Poznan University of Medical Sciences, Szpitalna Street 27/33, 60-572 Poznan, Poland; pkrzyzanowska@ump.edu.pl (P.K.-J.); mduszuchowska@ump.edu.pl (M.D.-Ż.); lkaluzny@ump.edu.pl (Ł.K.)

**Keywords:** inborn error of metabolism, phenylalanine, prothrombin induced by vitamin K absence, nutrition, diet, adherence, non-adherence

## Abstract

This is the first study to evaluate vitamin K status in relation to dietary intake and phenylalanine dietary compliance in patients with phenylketonuria (PKU). The dietary and PKU formula intake of vitamin K was calculated in 34 PKU patients, with vitamin K status determined by the measurement of prothrombin induced by vitamin K absence (PIVKA-II). Blood phenylalanine concentrations in the preceding 12 months were considered. There were significantly more phenylalanine results exceeding 6 mg/dL in patients with normal PIVKA-II concentrations than in those with abnormal PIVKA-II levels (*p* = 0.035). Similarly, a higher total intake of vitamin K and dietary vitamin intake expressed as μg/day (*p* = 0.033 for both) and %RDA (*p* = 0.0002 and *p* = 0.003, respectively) was observed in patients with normal PIVKA-II levels. Abnormal PIVKA-II concentrations were associated with a lower OR (0.1607; 95%CI: 0.0273–0.9445, *p* = 0.043) of having a median phenylalanine concentration higher than 6 mg/dL. In conclusion, vitamin K deficiency is not uncommon in phenylketonuria and may also occur in patients with adequate vitamin K intake. PKU patients with better dietary compliance have a higher risk of vitamin K deficiency. The present findings highlight the need for further studies to re-evaluate dietary recommendations regarding vitamin K intake, both concerning formula-based and dietary consumption of natural products.

## 1. Introduction

The standard treatment in classical phenylketonuria (PKU) is based on the elimination of high-protein food (meat, milk, bread, etc.), therefore food for special medical purposes (FSMP) containing no phenylalanine (Phe) and special low-protein food items (e.g., bread-lp, cheese-lp, milk-lp) are essential for therapy. The optimization of dietary treatment, not limited to the composition of the FSMP preparation itself, creates a crucial challenge in current PKU care [1,2]. Since the first introduction of Phe-free preparations into PKU therapy, their composition has continuously been modified and improved. Within the last few years, PKU FSMPs were also supplemented with selenium and docosahexaenoic acid. Despite all efforts undertaken to adapt medicinal products to the needs of PKU patients, and high micronutrient intake (median above 200% of the reference nutrient intakes-RNI), biochemical markers suggest that some deficiencies still occur (e.g., selenium, zinc) [3,4], highlighting the further need for the nutritional profiling of L-amino acid supplements in PKU. 

Within 50 years of its discovery, vitamin K attracted much less research attention than other fat-soluble vitamins [5]. However, over the last few decades, the perception of the role of vitamin K has changed significantly. Schwalfenberg appreciated the emerging group of vitamins (K1 and K2) and underlined their essential role in human health [6]. Indeed, vitamin K plays a significant role not only in endocrinology (osteoporosis) and cardiology (prevention of coronary vessels calcification and prophylaxis of cardiovascular diseases in general) but also in hematology and neurology [7,8,9]. Recent data suggest that a higher dietary vitamin K intake is significantly associated with a lower presence of depressive symptoms [10]. Furthermore, in patients with chronic kidney disease, adequate intake of vitamin K may be associated with reduced all-cause and cardiovascular disease mortality [11]. Vitamin K is also needed for the sphingolipid synthesis, an essential element of myelin and cellular membranes [12]. Since the symptoms of untreated PKU comprise alteration of myelin synthesis, adequate vitamin K intake by patients seems to have an essential role.

There have been reports on adequate or inadequate vitamin K intake in PKU patients on a diet [13,14,15]. Still, even in recent studies investigating possible vitamin and micronutrient deficits, the status of vitamin K is not addressed [16,17,18]. Our present report is, to the best of our knowledge, one of the very few studies to investigate the status of vitamin K in PKU patients and the first to evaluate vitamin K status in relation to dietary intake and Phe dietary compliance.

## 2. Material and Methods

The study was conducted in a group of classical PKU patients born between 1980–2015 treated at the Department of Paediatric Gastroenterology and Metabolic Diseases of the Poznan University of Medical Sciences and Department of Paediatrics, Research and Development Centre in Wrocław, two reference centres for PKU patients in western Poland. The study group comprised 34 patients with PKU, 11 male (32.3%) and 23 female (67.7%). For the purposes of our analysis, patients with classical PKU were defined as those who, at diagnosis, required a low Phe diet to maintain plasma Phe levels within the target range of 2–6 mg/dL (120–360 μmol/L) and whose Phe levels without diet exceeded 20 mg/dL (1200 μmol/L) [19]. The inclusion criteria were classical PKU diagnosed in the screening programme and continuous treatment. The exclusion criteria included chronic or acute disease, which may influence PKU treatment or vitamin K absorption/metabolism and pregnancy. The studied PKU group was followed by a multi-disciplinary team (medical doctor, dietician, nurse), with the patients undergoing physical examination, as well as measurement of body mass and height. 

All patients followed from birth the recommendations of Phe-restricted diet. All patients but one were on Phe-free formula during the present study. None of the patients was receiving additional treatment (eg, BH_4_ or PEG-PAL formulation). The diet of the studied patients was analysed based on nutritional records collected via a 3-day diary (two weekdays and one weekend day). In all PKU patients, the average content of vitamin K in daily food and PKU formula was calculated (TiqDiet, Spotbeans, Szczecin, Poland) and compared to the American recommendations of recommended daily allowance (RDA) [20,21]. For the most accurate assessment of vitamin K intake, our own database was used, based on a review of world literature on the level of consumption and content of vitamin K in food products and PKU formulas.

Vitamin K status was determined by the measurement of PIVKA-II (prothrombin induced by vitamin K absence) using the Asserachrom PIVKA-II immunoassay kit (DeCarboxy Prothrombin, Diagnostica Stago, Asnières-sur-Seine, France) as described previously [22,23]. The cut-off value was set at 3 ng/mL, with higher concentrations considered abnormal (vitamin K deficiency), whereas lower levels were considered normal.

All patients followed from birth the recommendations of routine checks of Phe concentrations. During the study, patients were recommended to draw blood weekly in the first year of life, fortnightly up to 12 years of age, and monthly after that [1]. All blood Phe concentrations collected during the 12 months preceding blood collection were evaluated in dry blood spot, a venous blood sample (0.5 mL to clot) taken before eating in the morning following an overnight fast was checked using a fluorometric method. The Phe results were expressed as milligram per decilitre (mg/dL). The percentage Phe concentration within the therapeutic range was calculated for each patient according to recent recommendations [1]. Similarly, the percentage Phe concentration exceeding the therapeutic range of 6 mg/dL (SPIKE 6) and its doubled value (SPIKE 12) was calculated.

All results are expressed as medians and interquartile ranges (IQRs) and means ± standard deviations (SD). The odds ratio (OR) was calculated to compare the patient’s risk of abnormal metabolic control between PKU patients with normal and abnormal vitamin K status (as defined by PIVKA-II concentrations). The 95% confidence interval (95% CI) was calculated to estimate the precision of the OR. The Mann–Whitney U Test was used to compare the concentrations and total vitamin K intake in patients with normal and abnormal PIVKA-II concentrations. Spearman’s rank correlation was used to measure the strength of the relationship between variables. The level of significance was set at *p* < 0.05 and statistical analysis was performed using STATISTICA v. 13.1 (TIBCO, Palo Alto, CA, USA).

The study design was compliant with the Helsinki Declaration of 1975 as revised in 2013 and was approved by the Bioethical Committee at the Poznan University of Medical Sciences (Poland) (approval number: 84/12, 693/12, 269/14). Written informed consent was obtained from all participants.

## 3. Results

The clinical characteristics of the study group are presented in Table 1.

Patients differed in both metabolic control and vitamin K intake and status. In the case of 16 (47.0%) PKU patients, Phe concentrations were abnormal in at least 40% of performed measurements. Within this patient group, 12 patients (35.3%) had abnormal Phe concentrations in at least 70% of the measurements. Eighteen subjects (52.9%) had more than 50% Phe measurements with values higher than 6 mg/dL. Vitamin K status (defined by PIVKA-II concentration) was normal in 25 patients (73.5%), whereas 32 subjects (94.1%) fulfilled the recommendations of sufficient vitamin K intake. 

There were significantly more SPIKEs 6 in patients with normal PIVKA-II concentrations than in those with abnormal levels (*p* = 0.035). Similarly, a higher total intake of vitamin K and dietary vitamin intake expressed as μg/day (*p* = 0.033 for both) and %RDA (*p* = 0.0002 and *p* = 0.003, respectively) was observed (Table 2) in patients with normal PIVKA-II levels. Abnormal PIVKA-II concentrations were associated with a lower OR (0.1607; 95%CI: 0.0273–0.9445, *p* = 0.043) of having a median Phe concentration higher than 6 mg/dL (calculated based on the previous twelve-month measurements).

Vitamin K status (PIVKA-II concentrations) was not associated significantly (rho = −0.1967) with Phe intake (calculated per kg of body weight). Similarly, the relationship between PIVKA-II levels and vitamin K intake (%RDA) did not reach the level of significance (rho = −0.2951, *p* = 0.090). However, a significant relationship between Phe intake (calculated per kg of body weight) and vitamin K intake (%RDA) was documented (rho = 0.4173, *p* < 0.014).

## 4. Discussion

This is the first study that documented vitamin K deficiency in a significant percentage of PKU patients, establishing a clear link between vitamin K status and metabolic control, as evidenced by Phe concentrations, which could be attributed to dietary compliance/non-compliance. The intake of vitamin K was lower, with a poorer vitamin K status in patients with better metabolic control. The difference in vitamin K consumption was related to dietary intake. Interestingly, most patients with abnormal PIVKA concentrations met the recommended level of vitamin K intake.

Ekin et al. [24] documented lower phylloquinone concentrations in their 30 PKU patients compared to 30 healthy peers. However, the authors did not assess the dietary and formula intake of vitamin K. It is also important to underline that their assessment comprised the measurement of vitamin K1 exclusively. Although in the present study vitamin K levels were not determined, we assessed concentrations of undercarboxylated prothrombin (PIVKA-II), which is produced in the case of vitamin K deficiency. The production of PIVKA-II is dependent on both vitamin K1 and K2 resources, representing a functional deficiency of vitamin K. In two out of our nine PKU patients with abnormal PIVKA-II levels, total vitamin K intake (both from diet and formula) did not reach recommended levels. However, in the remaining seven subjects, it was higher, and the median value accounted for 187.3% of the US RDA. 

Green et al. [15] assessed the nutritional intake of macro- and micro-nutrients in sixteen adherent and fourteen non-adherent patients. Contrary to our findings, they documented that vitamin K intake was lower in non-adherent subjects, but their observations concerned only the intake of vitamin K1. Commercial databases contain limited information on vitamin K product content, so in the present study, we collected all available data from different nutritional programmes for every food item and product consumed by our patients, as well as assessing both vitamin K1 and K2 consumption. Considering protein intake, which was lower in the non-adherent than adherent subjects studied by Green et al. [15], the non-adherence seems to be related to the very low use of FSMP and lp-products. This may be, at least in part, explained by the older age of the patients studied (in comparison to our patients). In our PKU subjects, the predominant mode of non-adherence was related to almost the same use of Phe-free/low containing products with additional consumption of forbidden food items.

Almost twenty-five years ago, Schulpiss et al. [25] suggested vitamin K deficiency in PKU patients based on examination of blood coagulation factors. They documented significantly lower factor VII and X concentrations in PKU patients with good compliance compared to subjects on a relaxed diet. The authors suggested that in the compliant group of PKU children, especially in a group consuming food rich in vegetables, the reduced fat intake might have impaired the absorption of vitamin K, thus influencing the synthesis of vitamin K-dependent haemostatic factors. As proof of their concept, they referred to lower low-density lipoprotein and cholesterol levels in PKU patients than in controls and lower cholesterol concentrations in compliant than in non-compliant subjects.

The recommended intake of vitamin K was based upon representative dietary intake data from healthy individuals. The adequate intake (AI) in the US for men and women was set at 120 and 90 μg/day, respectively [21]. The European Food Safety Authority (EFSA) suggested a slightly lower AI of 70 μg phylloquinone/day for both men and women in 2017 [26]. European vitamin K experts indicated that the requirements for vitamin K for functions other than blood coagulation might be higher and suggested a reassessment of the present recommended dietary allowance [11,27]. 

The major difference in vitamin K intake in non-adherent and adherent PKU patients in the present study was related to dietary intake (*p* = 0.0002), whereas the formula-based consumption was almost the same. The main source of vitamin K in food for adherent PKU patients is vitamin K1, which is contained in leafy green vegetables, broccoli, sauerkraut, oils and in margarine based on vegetable oils. However, patients who do not follow a diet because of snacking, i.e., taking non-permitted products (meat, dairy products, etc.), have more vitamin K2 from food. In the present study, both non-adherent and adherent PKU patients received almost the same amount of vitamin K1 from a low phenylalanine or phenylalanine-free formula; the major difference was related to dietary vitamin K intake, predominantly comprising vitamin K1. Different forms of vitamin K are differently absorbed, depending on their type. While the absorption of vitamin K1 reaches only 5–15% of its total food consumption, vitamin K2 is absorbed almost completely [25]. It may be one of the possible explanations of the more frequent occurrence of normal PIVKA-II values (<3 ng/mL), i.e., normal vitamin K stores, in non-compliant patients. 

Recently, there has been more emphasis on the coexistence of other diseases and abnormalities in PKU not only related to central nervous system involvement [28]. Burton et al. [29] documented that the highest adjusted prevalence ratio (PR) was for renal insufficiency with hypertension (PR 95%CI: 2.20 1.60–3.00; *p* < 0.0001). It has been suggested that non-compliance (Phe concentrations exceeding the upper limit of 360 μmol/L defined by the American College of Medical Genetics and Genomics), which is present in more than 60% and 70% of adolescents and adults with PKU in the US respectively, is responsible for a higher incidence of at least some comorbidities. A potential link between renal, as well as cardiovascular, diseases and vitamin K deficiency demands, due to its speculative character, further studies. 

The inference from the present study is limited due to its cross-sectional design, which makes it difficult to draw causality. We did not measure vitamin K concentrations (including phylloquinone and MK-4 and MK-7 isoforms of vitamin K2), rather, we measured PIVKA-II levels that reflect vitamin K deficiency and might be functionally informative and clinically more significant (with undercarboxylated prothrombin being both K1 and K2-dependent). Vitamin K deficiency definitely results in higher PIVKA-II levels. However, “higher” or “lower” normal vitamin K status does not result in respectively lower or higher PIVKA-II concentrations. Although it has been accepted that PIVKA-II determination is characterized by a higher sensitivity and specificity as compared to other methods traditionally used for the assessment of vitamin K status in newborns and adults [30], the development of new techniques allowing for the evaluation of vitamin K1, MK-4 and MK-7 levels may change the foregoing attitude. However, these methods are still expensive and hardly available (especially the determination of vitamin K2 isoforms). The range of PIVKA-II values differs depending upon the applied method [31]. Moreover, the primary interest in PIVKA-II use concentrates not on a cut-off level between values in vitamin K deficiency and normal vitamin K status but on the cut-off level for the detection of liver tumors. The prevalence of vitamin K deficiency, as assessed by PIVKA-II levels using ELISA (applied in the present study), is not fully known. There are various reports, from the one documenting a high percentage of abnormal PIVKA-II levels (36%) in the Alaskan Yup’ik population (quite often not consuming vitamin K-rich products) to a non-appearance of vitamin K deficiency in the control group of a recent Polish study [32,33,34]. Abnormal PIVKA-II concentrations were also documented in several diseases other than PKU [35,36,37]. Another problem is related to the different cut-off levels suggested in the past by different manufacturers, which makes the comparison difficult. 

The assessment of vitamin K dietary intake is challenging due to shortcomings in reliable information regarding phylloquinone and menaquinone content in different natural and some commercial products (both lack of information and discrepancies) [38,39,40]. Comparisons of food composition data published decades apart are not fully reliable, as there have been changes in data sources, crop varieties, geographic origin, ripeness, sample size, sampling methods, laboratory analysis and statistical treatment over time, which affect reported nutrient levels [41]. In an attempt to overcome these difficulties, we created our own database, including vitamin K1 content as well as vitamin K2. The strength of the present study is the detailed characteristics of the metabolic control as well as the simultaneous measurement of vitamin K intake and status. Calculation of Phe intake (in any form of dietary assessment) is less reliable in the evaluation of dietary compliance than the measurements of Phe levels (used in the present study), documenting more objectively what really happened.

In conclusion, vitamin K deficiency is not uncommon in phenylketonuria, and may also occur in patients with adequate vitamin K intake according to the recent recommendations. Interestingly, PKU patients with better dietary compliance presented a higher risk of vitamin K deficiency. The present findings demand further studies re-evaluating the dietary recommendations on vitamin K intake, both concerning formula-based and dietary consumption of natural products. 

## Figures and Tables

**Table 1:** Clinical characteristics of the study group (n=34).

Parameter	Age (Years)	Z-Score		Phe Concentrations		Blood Drawings (% of Recommended)	SPIKE 6 (%)	SPIKE 12 (%)	PIVKA II (ng/mL)	Phe Intake (mg/day)	Protein Intake		Total Vitamin K Intake	
		Body Height	Body Weight	Median (mg/dL)	Abnormal (%)						Total (g/day)	From Formula /kg Body Weight	(µg/day)	%RDA
Range	0.8–35.0	−2.05–1.47	−1.66–4.38	1.57–23.6	0–100	16.7–450.0	0–100	1.2–3.8	0–100	182–736	15.7–136.0	0–3.44	33.0–286.6	57.8–523.8
Median (IQR)	17.9 (6.1–24.0)	−0.13 (−0.60–0.58)	0.18 (−0.4–0.74)	6.8 (3.9–11.5)	30.0 (10.1–78.8)	83.3 (50.0–111.5)	63.9 (22.4–95.0)	2.3 (2.1–3.0)	5.5 (0–47.5)	362 (284–426)	60.4 (33.1–76.6)	1.06 (0.85–1.24)	125.1 (83.0–153.5)	158.0 (110.4–227.2)
Mean (SD)	16.3 (10.8)	−0.08 (0.83)	0.25 (1.13)	8.1 (5.4)	46.3 (39.1)	96.3 (86.3)	55.1 (36.4)	2.5 (0.7)	26.3 (35.3)	370 (120)	59.5 (30.0)	1.10 (0.57)	125.1 (58.7)	196.1 (113.0)

Phe—phenylalanine; SPIKE 6—the percentage of Phe concentrations exceeding the cut-off level of 6 mg/dL; SPIKE 12—the percentage of Phe concentrations exceeding the cut-off level of 12 mg/dL; PIVKA—prothrombin induced by vitamin K absence; RDA—recommended daily allowances; IQR—interquartile range; SD—standard deviation.

**Table 2:** The comparison of Phe concentrations and total vitamin K intake in patients with normal and abnormal PIVKA-II concentrations.

* PIVKA-II < 3 ng/mL (*n* = 25)								
Parameter	Phe Concentrations		SPIKE 6	SPIKE 12	Total Vitamin K Intake		Dietary Vitamin K Intake	
	Median (mg/dL)	Abnormal (%)	(%)	(%)	(µg/day)	%RDA **	(µg/day)	%RDA **
Range	1.6–23.6	0–100	5.8–100	0–100	33.0–255.0	85.4–523.8	17.0–154.3	31.4–463.8
Median (IQR)	7.8 (4.6–12.2)	66.7 (18.4–100)	71.4 (41.7–100)	7.4 (0–66.7)	131.0 (95.7–157.1)	187.3 (111.1–234.8)	70.9 (49.9–123.0)	88.7 (60.2–153.8)
Mean (SD)	9.0 (5.8)	52.0 (39.5)	63.7 (33.6)	30.9 (38.9)	135.9 (57.4)	210.2 (122.9)	80.7 (40.9)	134.2 (108.4)
* PIVKA-II > 3 ng/mL (*n* = 9)								
Range	2.7–11.6	0–100	0–100	0–50.0	56.2–155.9	57.9–333.1	15.3–55.9	24.1–76.6
Median (IQR)	4.3 (3.7–4.5)	22.2 (0–30.4)	23.1 (0–30.4)	5.4 (0–15.4)	82.3 (66.6–109.1)	136.3 (102.8–197.7)	28.9 (24.4–34.1)	56.0 (32.0–57.7)
Mean (SD)	5.5 (3.4)	30.3 (35.2)	31.2 (34.9)	13.4 (18.7)	90.9 (33.1)	156.9 (81.0)	30.8 (12.9)	50.1 (19.0)
*p*	0.101	0.207	0.035	0.549	0.033	0.263	0.0002	0.003

Phe—phenylalanine; PIVKA—prothrombin induced by vitamin K absence; SPIKE 6—the percentage of Phe concentrations exceeding the cut-off level of 6 mg/dL; SPIKE 12—the percentage of Phe concentrations exceeding the cut-off level of 12 mg/dL; RDA—recommended daily allowances; IQR—interquartile range; SD—standard deviation. * PIVKA-II concentrations lover than 3 ng/mL were considered as normal. ** US reference values.
